# Short sleep duration and interest in sleep improvement in a multi-ethnic cohort of diverse women participating in a community-based wellness intervention: an unmet need for improvement

**DOI:** 10.1186/s12905-023-02341-z

**Published:** 2023-04-20

**Authors:** Sara E. Simonsen, Grant R. Sunada, Kathleen Digre, Louisa A. Stark, Valentine Mukundente, Ed Napia, Fahina Tavake-Pasi, Jeannette Villalta, Doriena Lee, France Davis, Ana Sanchez-Birkhead, B. Heather Brown, Kelly G. Baron

**Affiliations:** 1 University of Utah College of Nursing, 10 South 2000 East Rm 4200, Salt Lake City, UT 84112 USA; 2 Department of Family and Preventive Medicine, Division of Public Health, 375 Chipeta Wy Suite A, Salt Lake City, Utah 84108 USA; 3 Departments of Neurology, Ophthalmology, and Obstetrics and Gynecology, University of Utah, 175 N Medical Dr E Rm 5001, Salt Lake City, UT 84132 USA; 4 University of Utah Genetic Science Learning Ctr, 515 E 100 S Rm 300, Salt Lake City, Utah 84112 USA; 5 Best of Africa, 6379 Thor Way, West Valley, Utah, 84128 USA; 6 Urban Indian Center of Salt Lake, 120 West 1300 South, Salt Lake City, Utah, 84115 USA; 7 National Tongan American Society, 3007 South West Temple #H, Salt Lake City, Utah 84115 USA; 8 Hispanic Health Care Task Force, 5280 Commerce Dr. Suite E140, Murray, Utah 84107 USA; 9 Calvary Baptist Church, 1090 South State Street, Salt Lake City, Utah, 84111 USA; 10 University of Utah Center for Clinical and Translational Science, 515 East 100 South, Salt Lake City, Utah, 84102 USA

**Keywords:** Short sleep, Disparities, Health behavior change, Community-engaged research, Obesity

## Abstract

**Background:**

Disparities in sleep duration are a modifiable contributor to increased risk for cardiometabolic disorders in communities of color. We examined the prevalence of short sleep duration and interest in improving sleep among a multi-ethnic sample of women participating in a culturally tailored wellness coaching program and discussed steps to engage communities in sleep health interventions.

**Methods:**

Secondary analysis of data from a randomized trial were used. The wellness coaching trial utilized a Community-Based Participatory Research (CBPR) approach. Data were from the baseline survey and baseline wellness coaching notes. Short sleep duration was defined as < 7 h of self-reported sleep. Participants were prompted to set a goal related to healthy eating/physical activity and had the opportunity to set another goal on any topic of interest. Those who set a goal related to improving sleep or who discussed a desire to improve sleep during coaching were classified as having an interest in sleep improvement. Analyses utilized multivariable models to evaluate factors contributing to short sleep and interest in sleep improvement. We present our process of discussing results with community leaders and health workers.

**Results:**

A total of 485 women of color participated in the study. Among these, 199 (41%) reported short sleep duration. In adjusted models, Blacks/African Americans and Native Hawaiians/Pacific Islanders had higher odds of reporting < 7 h of sleep than Hispanics/Latinas. Depression symptoms and self-reported stress management scores were significantly associated with short sleep duration. Interest in sleep improvement was noted in the wellness coaching notes of 52 women (10.7%); sleep was the most common focus of goals not related to healthy eating/physical activity. African Immigrants/Refugees and African Americans were less likely to report interest in sleep improvement. Community leaders and health workers reported lack of awareness of the role of sleep in health and discussed challenges to obtaining adequate sleep in their communities.

**Conclusion:**

Despite the high prevalence of short sleep duration, interest in sleep improvement was generally low. This study highlights a discrepancy between need and interest, and our process of community engagement, which can inform intervention development for addressing sleep duration among diverse women.

## Background

Sleeping less than the recommended 7 h per night for adults is a risk factor for developing chronic conditions, including obesity, hypertension, and type 2 diabetes, metabolic syndrome, and all cause death [1–5]. The prevalence of short sleep among working Americans has increased since 2010 [6]. It also is thought to be one factor contributing to racial/ethnic health disparities [7–13]. Although studies have documented disparities in sleep duration and disorders among communities of color, much of the sleep disparities literature has focused on comparing differences in sleep between Black/African Americans and Hispanics with non-Hispanic whites [14]. These studies have demonstrated that Black/African Americans are nearly twice as likely to have short sleep duration [14]. Rates of short sleep duration are also higher among Hispanics/Latinos compared to non-Hispanic whites [7]. Less is known about the prevalence of short sleep duration and its consequences for other diverse populations, such as Native Hawaiians and other Pacific Islanders (NHPI) [11] and American Indians/Alaska Natives (AI/AN) [15]. The National Health Interview Survey (2014) found a higher prevalence of short sleep duration in both NHPI and AI/AN respondents than in non-Hispanic white (NHW) and Hispanic/Latina participants; the NHPI and AI/AN rates were similar to those of Black/African American participants. For example, data analyses found that 40% of NHPI and 41% of AI/AN slept less than 7 h compared with 32% of NHW [16, 17]. This is consistent with data for other health risks, including increased risk for obesity, diabetes, and cardiovascular diseases in these groups.

Beyond documenting disparities, interventions are critically needed to improve health equity for communities of color [18], including for their sleep. The process of community-engaged research includes working with community members to identify needs and then design and implement interventions collaboratively [19]. To date, there have been few studies examining community perceptions and needs for sleep interventions, which is a major limiting factor to the development of sleep interventions among communities of color. Existing studies about sleep beliefs and practices have tended to focus on Black/African American communities [20–22]. Considering the high number of health and social issues that are disproportionately affecting communities of color, it is important to understand the need and desire for sleep-related interventions in additional communities. Another consideration for sleep interventions in communities of color is balancing sleep with other ongoing health concerns.

Our team recently completed a randomized trial of a 1-year community-based wellness intervention that enrolled a multi-ethnic sample and provided culturally tailored wellness coaching via community health coaches (The Coalition for a Healthier Community for Utah Women and Girls study, UWAG) [23–25]. The randomized trial utilized methods of Community-Based Participatory Research (CBPR) methods in all phases. In this paper we present information regarding sleep duration and interest in improving sleep among the women participating in our study. Second, we review our process of discussing the study findings among academic members, community leaders and community health workers (CHWs) in the research team in order to facilitate interpretation of findings and planning for next steps in this area of community-engaged research. This study provides important data regarding sleep needs and community perceptions about sleep among several understudied communities of color, including NHPI, AI/AN, and African Immigrants/Refugees.

## Participants and methods

### Participants and procedure

This is a secondary analysis of baseline data from the Coalition for a Healthier Community for Utah Women and Girls (UWAG) study, a CBPR-facilitated randomized behavioral trial assessing the effectiveness of a 12-month wellness coaching intervention on women’s physical activity levels and dietary habits [23] among five communities of color in Salt Lake City: Black/African Americans, Central African Immigrants/Refugees, Hispanics/Latinos, Native Americans/Alaska Natives (AI/AN) and Native Hawaiians/Pacific Islanders (NHPI). Most of the Central African Immigrants/Refugees arrived in the U.S. with official refugee status but consider themselves to be immigrants and prefer this terminology, thus we utilize the terms Immigrants/Refugees to refer to this group. Detailed information about UWAG study methods and the cost-effectiveness of the intervention have been published previously [23–25]. The UWAG study was planned and carried out in partnership with Community Faces of Utah (CFU), a coalition of leaders of the five diverse communities listed above; Utah Department of Health staff; and researchers from the University of Utah who collaborate in conducting multi-cultural, community-engaged research. With a shared commitment to CBPR, the Coalition conducted a needs assessment to identify priority issues, collaboratively identified an intervention, designed a clinical trial, trained CHWs from each community known as wellness coaches, conducted the trial, and contributed to data analysis and interpretation. Dissemination of study findings also follows a CBPR approach, with participation from community members throughout the process.

The women who participated in the UWAG study were recruited from the five CFU communities by CHWs. The study was described to potential participants as a trial of a wellness coaching intervention designed to address behaviors associated with obesity and chronic disease. Inclusion criteria for the study were: self-identification as a woman; self-identification as a member of one of the 5 target communities; age 18 or older; not currently participating in a wellness coaching program; fluent in English, Spanish, or Kirundi; willing to be randomized; willing to be followed for 12 months; and willing to complete interviews and have health data collected at baseline, 4, 8, and 12 months. We excluded individuals who were less than 18 years of age; currently participating in a wellness coaching program; were not fluent in English, Spanish, or Kirundi; were unwilling to be randomized; were unwilling to be followed for 12-months; or who were unwilling to participate in interviews and health data collection.

During the intervention, participants worked with a CHW from their own community to assess their health behaviors and set goals for health behavior changes over 12 months within a culturally tailored motivational interviewing framework. Wellness coaching occurred at baseline, 4-months, 8-months, and 12-months. This study utilizes secondary data analysis of baseline data. Focus areas of wellness coaching included healthy eating, physical activity, sleep duration, smoking cessation, hypertension, obesity, and mental health. Participants were encouraged to set a goal related to healthy eating/physical activity and were then invited to set a goal for another area of their choice. The study was designed to compare a low-intensity wellness coaching program (4 coaching sessions over 1 year) with a high-intensity program (monthly coaching over 1 year plus monthly group activities). Data on self-reported average hours of sleep were collected during each woman's initial baseline interview with her coach, and her interest in improving her sleep was documented during the coaching sessions.

All data used in the current analysis were collected at baseline and recorded in a REDCap database during the interview. Black/African American, NHPI, and AI/AN women were interviewed in English. Hispanic/Latina women had the option of either English or Spanish interviews, and African Immigrant/Refugee women were interviewed in English or Kirundi. Following the baseline interview, blood pressure and anthropometric measurements were taken, including height, weight (using an appropriately zeroed analogue scale), waist circumference, and hip circumference. After these measurements were taken, each participant received wellness coaching. Coaching sessions were in person and lasted 25 min on average. Each coaching session included a review of the woman’s self-reported health data and whether she was meeting health recommendations for fruit/vegetable servings per day, weekly minutes of physical activity, BMI, and hours of sleep per night. Recommendations for each of these areas were shared. Women reporting < 7 h of sleep were educated about the relationship between short sleep and heart disease, diabetes, depression, and obesity. The coaching session also included a review of each woman's depressions screening score and self-reported stress management score, measured using the wellness wheel described below. Participants were then prompted to set no more than 3 goals based on the information they had just received about their health, with a primary goal targeting healthy eating or physical activity (study’s primary outcomes) and additional goals, if desired, on any topic of interest.

All study procedures were approved by the University of Utah Institutional Review Board (IRB_00055195) and the Phoenix Area Indian Health Service Institutional Review Board. All procedures performed in studies involving human participants were in accordance with the ethical standards of the institutional and/or national research committee and with the 1964 Helsinki declaration and its later amendments or comparable ethical standards. This trial was also registered with clinical trials.gov (#NCT02470156). Written informed consent was obtained from all participants.

### Measures

#### Sleep

During the baseline interview, participants were asked, “On average, about how many hours do you sleep each night? (If respondent works a night shift, ask how many hours she sleeps during the day).” The data were recorded as discrete numbers and transformed into a dichotomous variable of (a) < 7 h and (b) 7 h or more.

#### Interest in sleep improvement

For the purpose of this study, interest in sleep improvement was defined as a participant setting a specific sleep-focused goal or identifying sleep as a focus area of improvement during the coaching session. The data come from text notes written by the wellness coaches, including notes describing specific goals and notes describing focus areas of interest.

#### Covariates, stress, and health behavior variables

Education and employment status were self-reported. Poverty status was calculated by comparing self-reported monthly income to the Federal Poverty Line (FPL) by year of survey and family size. Depression was measured using the Patient Health Questionnaire-2 (PHQ-2) [26], with scores greater than 3 out of 6 indicating a positive depression screen. Stress management scores and sleep wellness scores were measured using a wellness wheel with 4 dimensions of health (physical activity behaviors, eating behaviors, sleeping behaviors, and stress management behaviors) where participants were asked to identify their current health behaviors using a 0–10 scale. Higher scores indicate higher levels of stress management and higher levels of sleep wellness. Body mass index (BMI) was objectively measured by health coaches and calculated based on body weight (kg) / height (cm)^2^ with overweight ≥ 26 for NHPI and ≥ 25 for others [27, 28]. Fruit and vegetable intake questions were based on the Behavioral Risk Factor Surveillance System questions [29] (i.e., number of times 100% fruit juice, fruit, beans (legumes), dark green vegetables, orange vegetables, and other vegetables were consumed over the past month) and were stratified into 5 or more vs. less than 5 [30]. Physical activity was assessed with the following question, “In an average week, how much time do you spend being physically active or doing exercise?” [31]. The data were stratified into those engaging in the recommended 150 min per week or more vs. those engaging in less than 150 min per week [32].

### Data analysis

We first conducted analyses comparing the demographic and clinical factors with the self-report of < 7 h of sleep and interest in improving sleep using Chi-square test or Fisher's Exact test (when expected cell counts were < 5). Next, we used logistic regression models to examine predictors of sleep duration and interest in sleep interventions, to understand the role of mental health and physical comorbidities, which have a bidirectional association with sleep duration. For sleep duration, we conducted 2 separate models: 1) demographic variables and BMI and 2) added comorbidities, stress management score, and health behavior variables to the variables included in model 1. A series of logistic regression models were also used to evaluate predictors of interest in improving sleep during wellness coaching and covariates of interest. Model 1 included demographic variables and BMI and model 2 added sleep duration and self-assessed sleep wellness. When comparing communities, Hispanic/Latina participants were used as the comparison group as this was the largest group included in the study. Individuals with missing data on one or more variables were excluded from the respective multivariable model; data were missing for 0-7% of respondents, depending on the specific variable.

### Community-academic research team data review and interpretation process

As part of our CBPR process, the academic researchers analyzed the data and shared the findings with the community members on the research team. The entire team discussed how to interpret the data and foci for the discussion, with an emphasis on insights shared by community members [33] on the team. Additionally, study CHWs were asked to share their experiences working with women during the study, and their insights are included in the study results.

## Results

### Participant characteristics

A total of 485 women enrolled in the study and completed the baseline interview (Table 1). The racial/ethnic make-up of participants was 17% African Immigrant/Refugee, 21% Black/African American, 29% Hispanic/Latina, 18% NHPI, and 15% AI/AN. African Immigrants/Refugees were primarily from the central African countries of Rwanda, Burundi, and Congo. Most Hispanic/Latina women were from Mexico while some were born in the United States. A total of 43% of participants had a household income less than 100% FPL adjusted for household size. More than half of participants (58%) had some education beyond high school (including some college or graduation from college), and 35% had full-time employment. The health indicators in Table 1 suggest that study participants experience a number of health challenges; e.g., 83% were overweight or obese, with 56% being obese. When asked, "Do you currently have any of the following health problems?” 13% reported diabetes, 18% reported hypertension, and 9% reported sleep apnea. In addition, 22% had a positive PhQ-2 depression screen. A total of 71% of participants reported eating at least 5 fruits/vegetables per day, and 42% reported obtaining the recommended 150 min of physical activity per day (Table 1).

**Table 1:** Demographic and clinical characteristics of study participants

Variable	N (%) *N* = 485
**Age**	
18–34	180 (37.1%)
35–54	219 (45.2%)
55 +	86 (17.7%)
**Community**	
African Immigrant/Refugee	84 (17.3%)
Black/African American	102 (21.0%)
American Indian/Alaska Native	74 (15.3%)
Hispanic/Latina	138 (28.5%)
Native Hawaiian Pacific Islander	87 (17.9%)
**Education**	
< High school	75 (15.5%)
High school graduate	130 (26.8%)
> High school but not college graduate	183 (37.7%)
College graduate	97 (20.0%)
**Employed outside the home**	
Full-time	171 (35.3%)
Part-time, unemployed, other	305 (62.9%)
Missing	9
**Below federal poverty level**	210 (43.3%)
Missing	36
**BMI**	
**Mean (SD)**	32.2 (7.9)
Underweight/normal weight (< 25)	85 (17.5%)
Overweight (25–29.9)	128 (26.4%)
Obese (≥ 30)	272 (56.1%)
**Sleep apnea**	42 (8.7%)
**Diabetes**	59 (12.6%)
**Hypertension**	87 (17.9%)
**Positive PHQ-2 depression screen**	105 (21.6%)
**5 + fruits & vegetables/day**	346 (71.3%)
**150 + min physical activity/week**	
< 150 min physical activity	281 (57.9%)
150 + min physical activity	204 (42.1%)
**Self-reported sleep wellness – Mean (SD)**	6.50 (2.5)
**Self-reported stress management—Mean (SD)**	6.17 (2.5)
**Interest in Sleep Improvement**	57 (11.8%)

### Sleep duration

**Fig. 1:**
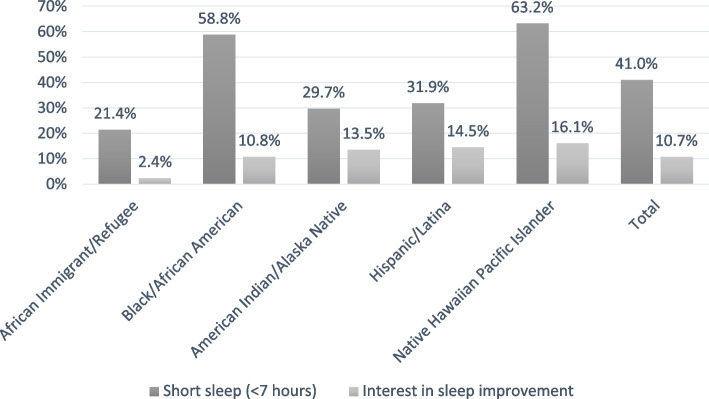
Frequency of short sleep (<7 hours) & interest in improving sleep among UWAG participants

The mean sleep duration for study participants was 6.8 h (SD = 1.46, range = 1–12, interquartile range = 6–8), and 41% of participants reported a sleep duration of < 7 h (Fig. 1). Based on results of unadjusted Chi-square test or Fisher's Exact test, where appropriate, there were statistically significant differences in short vs. recommended length of sleep by community, education level, employment status, BMI, self-reported sleep apnea, hypertension, positive PHQ-2 depression screen, self-reported stress management score, and self-reported sleep wellness (Table 2).

**Table 2:** Factors associated with short (< 7 h/night) and recommended length (≥ 7 h/night) sleep duration at baseline

Variable	Sleep duration < 7 h *N* = 199 (41.0%)	Sleep duration ≥ 7 h *N* = 286 (59.0%)	*P*
**Age**			0.447
18–34	69 (38.3%)	111 (61.7%)	
35–54	90 (41.1%)	129 (58.9%)	
55 +	40 (46.5%)	46 (53.5%)	
**Community**			**< 0.0001**
African Immigrant/Refugee	18 (21.4%)	66 (78.6%)	
Black/African American	60 (58.8%)	42 (41.2%)	
American Indian/Alaska Native	22 (29.7%)	52 (70.27%)	
Hispanic/Latina	44 (31.9%)	94 (68.1%)	
Native Hawaiian Pacific Islander	55 (63.2%)	32 (36.8%)	
**Education**			**0.009**
< High school	19 (25.3%)	56 (74.7%)	
High school graduate	51 (39.2%)	79 (60.8%)	
> High school but not college graduate	88 (48.1%)	95 (51.9%)	
College graduate	41 (42.3%)	56 (57.7%)	
**Employed outside the home**			**0.0203**
Full-time	82 (48.0%)	89 (52.1%)	
Part-time, unemployed, other	113 (37.1%)	192 (63.0%)	
Missing	4 (44.4%)	5 (55.6%)	
**Below federal poverty level**	78 (37.1%)	132 (62.9%)	0.070
Missing	12 (33.3%)	24 (66.7%)	
**BMI**			
Mean (SD)	34.1 (8.49)	30.9 (7.10)	**< 0.000**^**a**^
Underweight/normal weight (< 25)	27 (31.8%)	58 (68.2%)	**0.0028**
Overweight (25–29.9)	42 (32.8%)	86 (67.2%)	
Obese (≥ 30)	130 (47.8%)	142 (52.2%)	
**Sleep apnea**	24 (57.1%)	18 (42.9%)	**0.0263**
**Diabetes**	27 (45.8%)	32 (52.2%)	0.4305
**Hypertension**	44 (50.6%)	43 (49.4%)	**0.0472**
**Pos PHQ-2 depression screen**	54 (27.1%)	51 (17.8%)	**0.014**
**5 + fruits & vegetables/day**	143 (41.3%)	203 (58.7%)	0.833
**150 + min physical activity/week**			
< 150 min physical activity	116 (58.3%)	42 (60.9%)	0.908
150 + min physical activity	83 (41.7%)	244 (85.3%)	
**Self-reported sleep wellness—Mean, SD**	4.82 (2.1)	7.66 (2.0)	**< 0.0001**^**a**^
**Self-reported stress management—Mean, SD**	5.65 (2.5)	6.52 (2.5)	**0.0001**^**a**^
**Interest in Sleep Improvement**	46 (80.7%	11 (19.3%)	**< .0001**

Values in bold are statistically significant

^a^Wilcoxon Rank Sum (t approximation)

In adjusted models, those with a negative PHQ-2 depression screen were significantly less likely to report short sleep (OR = 0.47, 95% CI 0.26–0.84; Table 2). Higher stress management scores, indicating better levels of stress management, were significantly associated with lower odds of short sleep (OR = 0.86, 95% CI 0.78–0.94). There were no statistically significant associations between short sleep and BMI, age, employment, self-reported health conditions, diet, or exercise behaviors. In multivariable models, short sleep was significantly associated with being a member of the Black/African American (OR = 4.86, 95% CI 2.34–10.11) or NHPI (OR = 5.80, 95% CI 2.80–12.02) communities; this finding was consistent in both models (Table 3).

**Table 3:** Odds of short sleep duration (< 7 h) at baseline among women enrolled in UWAG

Variable	Crude Model: OR (95% CI) *N* = 485	Model 1: OR (95% CI) *N* = 440	Model 2: OR (95% CI) *N* = 439
**Community**			
African Immigrant/Refugee	0.58 (0.31–1.10)	0.79 (0.39–1.61)	0.78 (0.37–1.66)
Black/African American	**3.05 (1.79–5.20)**	**4.19 (2.14–8.19)**	**4.86 (2.34–10.11)**
American Indian/Alaska Native	0.90 (0.49–1.67)	0.97 (0.49–1.99)	0.93 (0.45–1.90)
Hispanic/Latina	REF	REF	REF
Native Hawaiian Pacific Islander	**3.67 (2.09–6.45)**	**4.61 (2.33–9.12)**	**5.80 (2.80–12.02)**
**BMI**			
vNormal		REF	REF
Overweight		1.07 (0.54–2.12)	1.019 (0.50–2.07)
Obese		1.48 (0.80–2.74)	1.43 (0.76–2.71)
**Age**			
18–34		REF	REF
35–54		1.32 (0.81–2.14)	1.34 (0.79–2.25)
55 +		1.21 (0.64–2.30)	1.35 (0.63–2.87)
**Federal poverty level**			
Above (vs Below)		1.64 (0.98–2.74)	1.67 (1.00–2.79)
**Employment Status**			
Fulltime (vs Not Full Time)		1.18 (0.75–1.86)	1.26 (0.78–2.03)
**Education**			
< High school		0.77 (0.37–1.61)	0.65 (0.30–1.44)
High school graduate		REF	REF
> High school/not college graduate		1.25 (0.73–2.14)	1.15 (0.65–2.03)
College graduate		0.76 (0.34–1.47)	0.77 (0.39–1.54)
**Sleep apnea**			1.56 (0.70–3.50)
**Hypertension**			1.06 (0.54–2.06)
**Diabetes**			0.65 (0.32–1.34)
**Depression Screening**			
Neg. PHQ-2 screen vs pos. screen			**0.47 (0.26–0.84)**
**Stress Self-Assessment**			
Self-reported stress management			**0.86 (0.78–0.94)**
**Fruit/Vegetable Intake**			
5 + fruits & vegetables/day vs < 5			1.31 (0.80–2.15)
**Physical Activity**			
150 + min physical activity/week vs < 150 min			0.91 (0.57–1.47)

Values in bold are statistically significant

### Interest in sleep improvement

Overall, 10.7% of participants (*n* = 52) discussed a desire to improve their sleep during baseline wellness coaching (Fig. 1). Sleep was the most common topic area for those who elected to set a goal unrelated to healthy eating and/or physical activity. Other goals were focused on stress management, mental health, blood pressure, smoking, and receipt of follow-up medical care. In bivariate analyses, there were statistically significant differences by the community, with NHPI women being the most likely to express interest in improving sleep (16.1%) and African Immigrants/Refugees being the least likely to report interest in improving sleep (2.4%). Individuals with diabetes were significantly more likely to identify a sleep focus (20.3% vs. 10.6%, *p* = 0.0289), and individuals consuming the recommended amount of fruits and vegetables per day were also more likely to have an interest in sleep improvement (20.1% vs. 8.4%, *p* = 0.0003) (Table 4). There were no statistically significant differences between a reported desire to improve sleep and other factors.

**Table 4:** Factors associated with interest in improving sleep after baseline wellness coaching among UWAG participants (*n* = 485)

One or More Sleep Goals	Interest in Improving Sleep *N* = 52 (10.7%)	No Interest in Improving Sleep *N* = 388 (89.3%)	*P*
**Age**			0.0859
18–34	14 (7.8%)	166 (92.2%)	
35–54	29 (13.2%)	190 (86.8%)	
55 +	14 (16.3%)	72 (83.7%)	
**Community**			**0.0160**
African Immigrant/Refugee	2 (2.4%)	82 (97.6%)	
Black/African American	11 (10.8%)	91 (89.2%)	
American Indian/Alaska Native	10 (13.5%)	64 (86.5%)	
Hispanic/Latina	20 (14.5%)	118 (85.5%)	
Native Hawaiian Pacific Islander	14 (16.1%)	73 (83.9%)	
**Education**			0.3882
< High school	5 (6.7%)	70 (93.3%)	
High school graduate	14 (10.8%)	116 (89.2%)	
> High school but not college graduate	26 (14.2%)	157 (85.8%)	
College graduate	12 (12.4%)	85 (87.6%)	
**Employed outside the home**			0.3327
Full-time	23 (13.5%)	148 (86.6%)	
Part-time, other and unemployed	32 (10.5%)	273 (89.5%)	
Missing	2 (22.2%)	7 (77.8%)	
**Federal poverty level**			0.0689
Below	19 (9.1%)	191 (91.0%)	
At or above	35 (14.6%)	204 (85.4%)	
**BMI**			
Mean (SD)	32.3 (7.87)	32.2 (7.86)	0.8888^a^
Underweight/normal weight (< 25)	9 (10.6%)	76 (89.4%)	0.9282
Overweight (25–29.9)	15 (11.7%)	113 (88.3%)	
Obese	33 (12.1%)	239 (87.9%)	
**Sleep apnea**	7 (16.7%)	35 (83.3%)	0.3013
**No sleep apnea**	50 (11.3%)	393 (88.7%)	
**Diabetes**	12 (20.3%)	47 (79.7%)	**0.0289**
**No diabetes**	45 (10.6%)	381 (89.4%)	
**Hypertension**	11 (12.6%)	76 (87.4%)	0.7757
**No hypertension**	46 (11.6%)	352 (88.4%)	
**Negative PHQ-2 depression screen**	46 (12.1%)	334 (87.9%)	**0.6464**
**Positive PHQ-2 depression screen**	11 (10.5%)	94 (89.5%)	
**Self-reported sleep wellness (**Mean (SD))	5.4 (2.27)	6.3 (2.53)	**0.0042**^**a**^
**Self-reported stress management (**Mean (SD))	4.6 (2.04)	6.7 (2.42)	**< .0001**^**a**^
**5 + fruits & vegetables/day**	28 (20.1%)	111 (79.9%)	**0.0003**
**< 5 fruits & vegetables/day**	29 (8.4%)	317 (91.6%)	
**Physical activity**			
< 150 min physical activity	27 (9.6%)	254 (90.4%)	0.0853
150 + min physical activity	30 (14.7%)	174 (85.3%)	

Values in bold are statistically significant

^a^Wilcoxon Rank Sum Test t approximation

In multivariate models (Table 5), African Immigrants/Refugees were significantly less likely to report interest in improving sleep (OR = 0.18 95% CI 0.03–0.94), as were Black/African Americans (OR = 0.24 95% CI 0.08–0.78), compared to Hispanic/Latina women. Those reporting short sleep were significantly more likely to report interest in improving sleep (OR = 5.70 95% CI 2.32–14.01), as were women reporting a diabetes diagnosis (OR = 2.99 95% CI 1.08–7.87), and those aged 35–54, compared to younger women, age 18–34 (OR = 2.44 95% CI 1.01–5.91). As levels of self-perceived sleep wellness improved, the likelihood of interest in improving sleep decreased (OR 0.81 95% CI 0.67–0.97). Those meeting the fruit/vegetable consumption guidelines or the physical activity guidelines were significantly more likely to report interest in improving sleep (OR 4.12 95% CI 1.96–8.64 and OR 2.42 95% CI 1.11–5.25, respectively).

**Table 5:** Odds of interest in improving sleep during baseline wellness coaching among UWAG participants

**Variable**	**Crude Model:** **OR (95% CI)** *N* = 485	**Model 1:** **OR (95% CI)** *N* = 440	**Model 3:** **OR (95% CI)** *N* = 439
**Community**			
African Immigrant/Refugee	**0.14 (0.03–0.63)**	**0.20 (0.04–0.94)**	**0.18 (0.03–0.94)**
Black/African American	0.71 (0.33–1.56)	0.71 (0.28–1.78)	**0.24 (0.08–0.78)**
American Indian/Alaska Native	0.92 (0.41–2.09)	0.83 (0.32–2.11)	0.57 (0.19–1.78)
Hispanic/Latina	REF	REF	REF
Pacific Islander	1.13 (0.54–2.38)	1.29 (0.52–3.18)	0.46 (0.14–1.49)
**Short sleep**			
< 7 h vs. 7 + hours			**5.70 (2.32–14.01)**
**BMI**			
Normal/underweight		REF	REF
Overweight		0.76 (0.26–2.16)	0.84 (0.27–2.60)
Obese		0.54 (0.21–1.41)	0.52 (0.18–1.49)
**Age**			
18–34		REF	REF
35–54		**2.32 (1.04–5.17)**	**2.44 (1.01–5.91)**
55 +		2.41 (0.88–6.62)	2.09 (0.64–6.88)
**Federal poverty level**			
Above (vs Below)		0.57 (0.26–1.26)	0.42 (0.18–1.03)
**Employment Status**			
Fulltime (vs Not Full Time)		1.16 (0.60–2.27)	1.54 (0.73–3.23)
**Education**			
< High school		1.11 (0.32–3.86)	1.35 (0.35–5.18)
High school graduate		REF	REF
> High school/not college grad		1.08 (0.47–2.49)	1.18 (0.46–3.03)
College graduate		1.00 (0.37–2.66)	0.89 (0.30–2.60)
**Sleep apnea**			1.20 (0.39–3.73)
**Hypertension**			0.94 (0.31–2.63)
**Diabetes**			**2.99 (1.08–7.87)**
**Depression Screening**			
Neg. PHQ-2 screen vs pos. screen			2.61 (0.98–6.96)
**Stress Self-Assessment**			
Self-reported stress management			0.87 (0.74–1.02)
**Sleep Self-Assessment**			
Self-reported sleep wellness			**0.81 (0.67–0.97)**
**Fruit/Vegetable Intake**			
5 + fruits & vegetables/day vs < 5			**4.12 (1.96–8.64)**
**Physical Activity**			
150 + min physical activity/week vs < 150 min			**2.42 (1.11–5.25)**

Values in bold are statistically significant

### Discussions among academics, community leaders and CHW study team members

As a part of our community-engaged research process, the academic partners shared the data analyses with the community leader partners and CHWs who worked on this project. Community leaders noted that the findings highlight a mismatch between awareness of the importance of sleep and lack of awareness of sleep guidelines in their communities. They were also interested in the connections between sleep with overweight and diabetes, which is a high priority health target in their communities. Leaders also mentioned that future sleep intervention work with their communities should consider traditional sleep patterns/norms that may vary across groups and impact current sleep behaviors. Study CHWs echoed the leaders’ statements that many of the study participants had not thought about sleep as a health issue before the study. One CHW described the discussion of sleep as a “wellness moment” during coaching, in which one of the study participants expressed surprise and shared that they had not been aware of this previously. Coaches reported that some participants were receptive to focusing on sleep improvement, while others felt that improving sleep was not possible due to competing demands, including work and caring for their families. CHWs expressed the importance of “meeting women where they are” during wellness coaching, helping them to focus on sleep health behavior changes that fit in with their needs and priorities.

## Discussion

### Short sleep and interest in sleep improvement

This study provides information regarding sleep duration and interest in sleep improvement using secondary baseline data from women in five communities of color who were participants in a randomized trial of a culturally tailored wellness coaching intervention. On average, the prevalence of short sleep duration in our study was higher than that reported in national surveys overall and in Black/African Americans and Pacific Islanders [28, 34]. In adjusted models, we found significant differences in the prevalence of short sleep between communities, with approximately fivefold higher rates of short sleep duration among Black/African American and NHPI women compared to Hispanic/Latina women. As would be expected, depressive symptoms and poorer self-reported stress management scores were associated with short sleep. Other studies have found similar associations between depression, stress, and short sleep [35, 36].

The design of our wellness intervention allowed us to examine participants’ interest in improving sleep, within the context of a multiple behavior change intervention. Despite a high prevalence of short sleep in most groups, there were low levels of prioritization of sleep behavior change in the context of wellness coaching related to multiple health behaviors; just over 10% of women indicated a desire to improve sleep. Because participants were asked to identify no more than 3 areas of health behavior change during their baseline wellness coaching session which emphasized a number of health behaviors, this percentage does not mean that sleep was not of interest, but indicates that it was viewed as being lower priority than other health behaviors. It is important to note that all participants were prompted to set a goal related to healthy eating or physical activity and invited to set an additional goal of their choosing. All participants set at least one goal. Sleep was the most frequently selected topic among those who set a secondary goal not related to healthy eating/physical activity.

Individuals with diabetes were more likely to be interested in improving sleep, which may be due to an increased awareness of their health and the need to make changes in order to improve or maintain it in the face of disease. Furthermore, individuals who were meeting the fruit and vegetable consumption guidelines or physical activity guidelines were also more interested in improving sleep, which may mean that health-conscious individuals who already follow recommended diet and exercise guidelines may be more open to adding another health-improving behavior when it is brought to their attention or perhaps can focus on sleep because other behaviors are already established. Choosing sleep goals may have been a lower priority area for individuals who need improvement in their nutrition or physical activity behaviors.

Involving community leader research partners in interpreting results provided a deeper understanding of how the study findings fit within each community's unique cultural and social context. Community leaders expressed interest in further exploring the relationship between obesity, diabetes and short sleep. Another important area brought up by the leaders and CHWs was the differences in sleep practices among individuals in different communities. These include different cultural views of sleep, such as traditional sleep schedules and family sleeping arrangements [37–39]. Incorporating community-centered beliefs and practices was also identified as an important area to include in future research collaborations with these communities.

The results of our study, the lessons learned while conducting the study, and the rich discussions about the findings among the academic and community partners and the CHWs will serve as a foundation for future sleep health interventions. The wellness coaching intervention for the UWAG study was developed in collaboration with the CFU community leaders and was implemented across all five communities with a structured but flexible approach designed to address the unique cultural needs and preferences of each community. Our results also highlight the need for and the challenge in integrating sleep into health behavior change programs that address multiple health behavior change areas.

### Strengths and limitations

Strengths of this study include the large multi-ethnic cohort, the use of standardized measures to collect anthropometric and health behavior data, and the real world CBPR approach utilized throughout the study. Additionally, community collaboration on this manuscript facilitated a unique, community-focused interpretation of study findings. Limitations include use of secondary cross-sectional data, use of self-reported sleep duration, and lack of more nuanced sleep measures, such as validated insomnia questionnaires. Participants were asked about their sleep “on average” and were not asked about weekday and weekend sleep separately. Information was not collected on bed partners, day shift vs. night shift work, or other measures of sleep health. While we adjusted our models using available data, there were some important factors that we were unable to adjust for. These include time in the U.S., reproductive stage (e.g. menopausal status), experiences of discrimination, post-traumatic stress disorder, and current status as refugee vs. immigrant, among other variables. We used measures of self-reported stress management and sleep wellness based on a 0–10 scale, but did not include a validated measure of stress. The vast majority of our participants were overweight/obese (82.5%) so we did not restrict by BMI nor did we restrict analyses to the 91.3% of participants who did not self-report sleep apnea. Given the high prevalence of overweight/obesity in the study and the low prevalence of sleep apnea, is likely that sleep apnea is vastly undiagnosed and untreated among study participants. Future work should formally assess for sleep apnea, refer those who screen positive for treatment, and stratify results by those with and without sleep apnea. This study was not specifically designed to measure or change sleep; sleep was one of several health behaviors available in the intervention. Anecdotal feedback from the CHWs participating in our study indicated there was variability in coach enthusiasm about sleep improvement, and therefore some participants may have received more information or had a greater opportunity to discuss sleep with their coach. Despite these limitations, this paper addresses a gap in the literature by providing information on sleep in women from several understudied communities, highlighting disparities and areas for future research and moving ahead our process of addressing the sleep issues among communities of color in our region.

## Conclusions

In summary, the high prevalence of short sleep duration, as well as high prevalence of sleep-related health conditions (e.g., obesity and diabetes and depression) among the communities participating in our study, suggest the need for continued community-engaged sleep-focused research, particularly among Black/African Americans and NHPI communities. A community-engaged approach to this work will ensure that future studies address community priorities and collect data relevant to community members. Based on the findings from this study and lessons learned, academic researchers, community leaders and CHW’s will continue our collaboration in order to address sleep and related health outcomes in these communities. Next steps will include discussions about potential focus areas and subsequent development of culturally appropriate sleep interventions within the context of promoting wellness for communities of color.

## Data Availability

This trial was registered June 12, 2015 with clinical trials.gov (#NCT02470156). The datasets generated and analyzed during the current study are not publicly available due to privacy concerns but are available from the corresponding author on reasonable request.

## Abbreviations

AI/AN: American Indians/Alaska Natives
BMI: Body Mass Index
CBPR: Community-Based Participatory Research
CFU: Community Faces of Utah
CHW: Community Health Worker
FPL: Federal Poverty Line
NHPI: Native Hawaiians and other Pacific Islanders
NHW: Non-Hispanic white
PHQ-2: Patient Health Questionnaire-2
UWAG: The Coalition for a Healthier Community for Utah Women and Girls study

## References

[1] Besedovsky L, Lange T, Haack M (2019). The sleep-immune crosstalk in health and disease. Physiol Rev.

[2] Tobaldini E, Costantino G, Solbiati M, Cogliati C, Kara T, Nobili L (2017). Sleep, sleep deprivation, autonomic nervous system and cardiovascular diseases. Neurosci Biobehav Rev.

[3] Watson NF, Badr MS, Belenky G, Bliwise DL, Buxton OM, Buysse D (2015). Recommended amount of sleep for a healthy adult: a joint consensus statement of the American academy of sleep medicine and sleep research society. Sleep.

[4] Smiley A, King D, Bidulescu A. The Association between sleep duration and metabolic syndrome: The NHANES 2013/2014. Nutrients. 2019;11(11). 10.3390/nu11112582. 10.3390/nu11112582 PMC6893635 31717770

[5] Okoro CA, Courtney-Long E, Cyrus AC, Zhao G, Wheaton AG (2020). Self-reported short sleep duration among US adults by disability status and functional disability type: Results from the 2016 Behavioral Risk Factor Surveillance System. Disabil Health J.

[6] Khubchandani J, Price JH (2020). Short sleep duration in working American adults, 2010–2018. J Community Health.

[7] Cunningham TJ, Wheaton AG, Ford ES, Croft JB (2016). Racial/ethnic disparities in self-reported short sleep duration among US-born and foreign-born adults. Ethn Health.

[8] Jackson CL, Redline S, Kawachi I, Hu FB (2013). Association between sleep duration and diabetes in black and white adults. Diabetes Care.

[9] Jackson CL, Powell-Wiley TM, Gaston SA, Andrews MR, Tamura K, Ramos A (2020). Racial/ethnic disparities in sleep health and potential interventions among women in the United States. J Womens Health (Larchmt).

[10] Jackson CL, Walker JR, Brown MK, Das R, Jones NL. A workshop report on the causes and consequences of sleep health disparities. Sleep. 2020;43(8). 10.1093/sleep/zsaa037. 10.1093/sleep/zsaa037 PMC7420527 32154560

[11] Matthews EE, Li C, Long CR, Narcisse MR, Martin BC, McElfish PA (2018). Sleep deficiency among Native Hawaiian/Pacific Islander, Black, and White Americans and the association with cardiometabolic diseases: analysis of the National Health Interview Survey Data. Sleep Health.

[12] Jackson CL, Redline S, Emmons KM (2015). Sleep as a potential fundamental contributor to disparities in cardiovascular health. Annu Rev Public Health.

[13] Petrov ME, Long DL, Grandner MA, MacDonald LA, Cribbet MR, Robbins R (2020). Racial differences in sleep duration intersect with sex, socioeconomic status, and U.S. geographic region: the REGARDS study. Sleep Health.

[14] Petrov ME, Lichstein KL (2016). Differences in sleep between black and white adults: An update and future directions. Sleep Med.

[15] Nuyujukian DS, Anton-Culver H, Manson SM, Jiang L (2019). Associations of sleep duration with cardiometabolic outcomes in American Indians and Alaska Natives and other race/ethnicities: results from the BRFSS. Sleep Health.

[16] Nuyujukian DS, Beals J, Huang H, Johnson A, Bullock A, Manson SM (2016). Sleep duration and diabetes risk in American Indian and Alaska Native participants of a lifestyle intervention project. Sleep.

[17] Young MC, Gerber MW, Ash T, Horan CM, Taveras EM. Neighborhood social cohesion and sleep outcomes in the Native Hawaiian and Pacific Islander National Health Interview Survey. Sleep. 2018;41(9). 10.1093/sleep/zsy097. 10.1093/sleep/zsy097 29771373

[18] Srinivasan S, Williams SD (2014). Transitioning from health disparities to a health equity research agenda: the time is now. Public Health Rep.

[19] Eder MM, Evans E, Funes M, Hong H, Reuter K, Ahmed S (2018). Defining and measuring community engagement and community-engaged research: Clinical and translational science institutional practices. Prog Community Health Partnersh.

[20] Baron KG, Gilyard SG, Williams JL, Lindich D, Koralnik L, Lynch EB (2019). Sleep-related attitudes, beliefs, and practices among an urban-dwelling African American community: a qualitative study. Sleep Health.

[21] Nam S, Whittemore R, Jung S, Latkin C, Kershaw T, Redeker NS (2018). Physical neighborhood and social environment, beliefs about sleep, sleep hygiene behaviors, and sleep quality among African Americans. Sleep Health.

[22] Grandner MA, Patel NP, Jean-Louis G, Jackson N, Gehrman PR, Perlis ML (2013). Sleep-related behaviors and beliefs associated with race/ethnicity in women. J Natl Med Assoc.

[23] Buder I, Zick C, Waitzman N, Simonsen S, Sunada G, Digre K (2018). It takes a village coach: Cost-effectiveness of an intervention to improve diet and physical activity among minority women. J Phys Act Health.

[24] Simonsen SE, Ralls B, Guymon A, Garrett T, Eisenman P, Villalta J (2017). Addressing health disparities from within the community: Community-based participatory research and community health worker policy initiatives using a gender-based approach. Womens Health Issues.

[25] Simonsen SE, Digre KB, Ralls B, Mukundente V, Davis FA, Rickard S (2015). A gender-based approach to developing a healthy lifestyle and healthy weight intervention for diverse Utah women. Eval Program Plann.

[26] Kroenke K, Spitzer RL, Williams JB (2003). The Patient Health Questionnaire-2: Validity of a two-item depression screener. Med Care.

[27] World Health Organization. Regional Office for the Western Pacific. The Asia-Pacific perspective: redefining obesity and its treatment. Sydney: Health Communications Australia; 2000. https://apps.who.int/iris/handle/10665/206936.

[28] Hsu WC, Boyko EJ, Fujimoto WY, Kanaya A, Karmally W, Karter A (2012). Pathophysiologic differences among Asians, native Hawaiians, and other Pacific Islanders and treatment implications. Diabetes Care.

[29] Centers for Disease Control and Prevention. Surveillance of fruit and vegetable intake using the Behavioral Risk Factor Surveillance System. In: Department of Health and Human Services, editor. Atlanta; https://www.cdc.gov/brfss/pdf/fruits_vegetables.pdf.

[30] Wang X, Ouyang Y, Liu J, Zhu M, Zhao G, Bao W (2014). Fruit and vegetable consumption and mortality from all causes, cardiovascular disease, and cancer: Systematic review and dose-response meta-analysis of prospective cohort studies. BMJ..

[31] Health Resources and Services Administration. My bright future: Physical activity and healthy eating for adult women. In: Department of Health and Human Services, editor. Washington, D.C. 2013. https://naldc.nal.usda.gov/download/1759343/PDF.

[32] Piercy KL, Troiano RP, Ballard RM, Carlson SA, Fulton JE, Galuska DA (2018). The physical activity guidelines for Americans. JAMA.

[33] Bordeaux BC, Wiley C, Tandon SD, Horowitz CR, Brown PB, Bass EB (2007). Guidelines for writing manuscripts about community-based participatory research for peer-reviewed journals. Prog Community Health Partnersh.

[34] Centers for Disease Control and Prevention. Data and statistics: Short sleep duration among US adults. 2017. https://www.cdc.gov/sleep/data_statistics.html.

[35] McEwen BS, Karatsoreos IN (2015). Sleep deprivation and circadian disruption: Stress, allostasis, and allostatic load. Sleep Med Clin.

[36] Johnson DA, Lisabeth L, Lewis TT, Sims M, Hickson DA, Samdarshi T (2016). The contribution of psychosocial stressors to sleep among African Americans in the Jackson Heart Study. Sleep.

[37] Worthman CM, Brown RA (2013). Sleep budgets in a globalizing world: Biocultural interactions influence sleep sufficiency among Egyptian families. Soc Sci Med.

[38] Smit AN, Broesch T, Siegel JM, Mistlberger RE (2019). Sleep timing and duration in indigenous villages with and without electric lighting on Tanna Island, Vanuatu. Sci Rep.

[39] de la Iglesia HO, Fernández-Duque E, Golombek DA (2015). Access to electric light is associated with shorter sleep duration in a traditionally hunter-gatherer community. J Biol Rhythms.

